# Evaluation of a large-scale health department naloxone distribution program: Per capita naloxone distribution and overdose morality

**DOI:** 10.1371/journal.pone.0289959

**Published:** 2023-08-11

**Authors:** Caroline E. Freiermuth, Rachel M. Ancona, Jennifer L. Brown, Brittany E. Punches, Shawn A. Ryan, Tim Ingram, Michael S. Lyons

**Affiliations:** 1 Department of Emergency Medicine, University of Cincinnati College of Medicine, Cincinnati, Ohio, United States of America; 2 Center for Addiction Research, University of Cincinnati College of Medicine, Cincinnati, Ohio, United States of America; 3 Department of Emergency Medicine, Washington University, St Louis, Missouri, United States of America; 4 Department of Psychological Sciences, Purdue University, West Lafayette, Indiana, United States of America; 5 College of Nursing, The Ohio State University, Columbus, Ohio, United States of America; 6 Department of Emergency Medicine, The Ohio State University College of Medicine, Columbus, Ohio, United States of America; 7 Brightview Health LLC, Cincinnati, Ohio, United States of America; 8 Hamilton County Public Health, Cincinnati, Ohio, United States of America; 9 Department of Environmental and Public Health Sciences, University of Cincinnati College of Medicine, Cincinnati, Ohio, United States of America; Massachusetts General Hospital, UNITED STATES

## Abstract

**Objectives:**

To report per-capita distribution of take-home naloxone to lay bystanders and evaluate changes in opioid overdose mortality in the county over time.

**Methods:**

Hamilton County Public Health in southwestern Ohio led the program from Oct 2017-Dec 2019. Analyses included all cartons distributed within Hamilton County or in surrounding counties to people who reported a home address within Hamilton County. Per capita distribution was estimated using publicly available census data. Opioid overdose mortality was compared between the period before (Oct 2015-Sep 2017) and during (Oct 2017-Sep 2019) the program.

**Results:**

A total of 10,416 cartons were included for analyses, with a total per capita distribution of 1,275 cartons per 100,000 county residents (average annual rate of 588/100,000). Median monthly opioid overdose mortality in the two years before (28 persons, 95% CI 25–31) and during (26, 95% CI 23–28) the program did not differ significantly.

**Conclusions:**

Massive and rapid naloxone distribution to lay bystanders is feasible. Even large-scale take-home naloxone distribution may not substantially reduce opioid overdose mortality rates.

## Introduction

The highest ever number of opioid-related overdose deaths (OOD) in the U.S. was recorded in 2020 at 68,630, a 68% increase in just two years; age-adjusted synthetic OOD increased more than 1000% in the past decade to 11.4/100,000 [1, 2]. In 2017, Ohio ranked second in the United States in number of OOD, at a rate of 39.2 per 100,000 population [3]. Naloxone can rapidly reverse otherwise fatal opioid-induced respiratory depression [4]. However, the time window for efficacious administration is often less than time elapsed from overdose identification to emergency medical services arrival [5]. Community overdose education and naloxone distribution is a supported strategy to increase the chance that lay bystanders recognize an overdose victim and administer naloxone in time [6–10]. Although there are no scientific data to estimate how often a victim would survive when a bystander does not administer naloxone, survival after field administration is generally considered equivalent to a life saved [11, 12]. This assertion is supported by mechanism of drug action, the experience of emergency medical service providers, and the preliminary association between take-home naloxone (THN) programs and reductions in opioid-related overdose mortality [13, 14].

Despite growing acceptance of THN as an effective harm-reduction strategy and demonstration that naloxone can be administered by laypeople [6, 14, 15, 16], there is little evidence to define an ideal strategy for community distribution or the saturation point at which enough naloxone has been distributed to realize the maximum possible decrease in overdose deaths. Walley *et al*. suggested a dose response effect between distribution and mortality with the highest volume strata greater than 100 distributions per 100,000 population [17]. Bird *et al*. recommend distributing naloxone to 10 to 20 times more at-risk individuals than the number of annual OOD [18]. Most recently, Irvine *et al*. used modeling to suggest that the amount of naloxone distribution needed to achieve 80% usage in witnessed overdoses is variable, with a maximum estimated need of 1,270 kits per 100,000 population, although these numbers are affected by type of drug used and how naloxone was procured [19]. At a minimum, the scale of distribution thus far has been small relative to community population size and distributing to only those at-risk of overdose misses the far larger population of individuals who are not at risk but may nonetheless encounter an overdose victim [20, 21].

This manuscript retrospectively evaluates a naloxone distribution effort led by a county health department, with partners including local health systems, foundations, substance use treatment centers, correctional facilities, and community service agencies. The central aim was to rapidly distribute as much naloxone as possible to lay bystanders in the hope that saturating the surrounding county with THN would lead to an immediate and recognizable decline in OOD. The primary objective was to measure the number of naloxone cartons distributed per 100,000 persons in the county. Secondary objectives were to describe the program’s approach and populations served and to report the change in county OOD over time.

## Methods

Retrospective use of available data for this program evaluation of THN distribution was approved by the University of Cincinnati IRB, which granted a waiver of consent for participants. No verbal or written consent was obtained from participants who accepted naloxone and completed a data form.

### Take home naloxone (THN) distribution program

Hamilton County Public Health (HCPH) in Cincinnati, Ohio led this naloxone distribution program, named the Naloxone Distribution Collaborative (NDC), from October 2017 through December 2019. Prior to program launch, there was minimal distribution of THN to interested individuals within the county.

HCPH received a total of 28,193 donated cartons of naloxone, each containing two nasal naloxone spray bottles (two doses), 4 mg per bottle. Cartons were distributed regionally, with a majority of distribution sites located within the county of interest.

The health department managed inventory, recruited distribution sites, and distributed cartons directly through community events and health department service locations (e.g., syringe service program, local jail). Partnering sites (e.g., facilities offering services to people with opioid use disorder (OUD), other social service agencies, local businesses, faith-based organizations) were chosen based on their interactions with persons at risk for opioid overdose, people who may interact with those at risk for opioid overdose, community involvement, and willingness to participate. Sites then distributed directly to individuals and/or kept stock on hand for overdoses occurring at that location. Training regarding use of the naloxone was provided by HCPH employees upon distribution, utilizing a train the trainer method whereby sites could provide the training to those individuals ultimately receiving the naloxone.

Data forms were developed by the local health department in consultation with the evaluation team (see S1 Appendix). Basic demographic information, including name and address, was collected in accordance with the state board of pharmacy regulations. In addition, forms elicited intended reasons for use of naloxone, use of intravenous drugs by the individual, prior use of naloxone, overdose history and treatment history for OUD. Sites were instructed to complete a data form for each instance in which naloxone was provided to an individual, with a space to indicate the number of cartons distributed to each individual. These could be completed through a REDCap website interface or on paper for later data entry by health department staff. The program explicitly emphasized successful distribution over rigorous data collection, so compliance with form completion, while encouraged, was at staff and client discretion. Cartons distributed by local health systems did not have data forms completed to avoid unacceptable burden to healthcare staff.

## Analyses

### Selection of participants

Cartons were included in analyses if there was documentation of: i) distribution occurring to an individual from a site in Hamilton County, or ii) distribution to an individual with a reported residence within Hamilton County. Individuals were not viable as a primary unit of analysis, as data were insufficient to reliably account for repeat encounters or to determine number of cartons provided to each individual.

### Data sources

HCPH maintained inventory logs tracking provision of naloxone cartons to distribution sites. Individual carton recipient information was obtained from the program-specific database (distributions that occurred outside of the health systems) and from electronic health record query (distributions from health systems). Only one of the four participating health systems provided data from the electronic health record, which was limited to basic demographic and residential information routinely collected for clinical care. U.S. census data was used for county population. The county’s population mid-year 2018 was 816,684 [22]. Data regarding naloxone prescriptions filled within the county was provided by Emergent Biosolutions (formerly Adapt Pharma). The local health department queried surveillance records for: unintentional (i.e., excluding those identified as suicide) OOD for individuals. Residential address was used to assign county at the time of death.

### Statistical analysis

The primary outcome was per capita distribution (number of cartons distributed per 100,000 county residents, overall and an average annual rate). For the primary analysis we used cartons as a surrogate measure for individuals.

Secondary outcomes included: i) categories of distribution sites or events, ii) self-reported recipient characteristics including reasons for interest in THN, expected use of THN, and opioid-overdose risk and history, and iii) opioid-related overdose deaths. For self-reported recipient characteristics, we used the distribution encounter as the unit of analysis. Data quality is insufficient in terms of both accuracy and availability to allow for a deduplicated analysis using unique individuals as the unit of analysis. This is because any individual may have participated in the NDC program more than once (i.e., multiple encounters) and/or may have received more than one carton of THN at any given encounter. In addition, a number of recipients used names thought to be other than their own.

#### Analysis was primarily descriptive

The outcome of OOD was measured by overall percent decrease, using a one-sided Wilcoxon rank-sum to test if median monthly rates of OOD decreased significantly between the two years prior to the program (October 2015—September 2017) and the two years during the program (October 2017—September 2019). Bootstrapping (N = 1000) was used to calculate median 95% confidence intervals (CIs). The trend line and 95% CI band for monthly OOD were calculated using LOESS (locally estimated scatterplot smoothing) regression due to the nonparametric nature of the data. We used negative binomial regression during the two years prior to the program to predict the number of monthly OODs for the study period. R (version 4.0.2) was used for all analyses [23]. DescTools package (version 0.99.44) was used to calculate the CIs for the medians [24]. Change in OOD was additionally evaluated for neighboring and similar counties within the state during the same time period for public health context.

## Results

Of the 28,193 cartons provided to the county health department, 17,777 were either allocated for first responder administration, distributed outside of the county to non-county residents or were unaccounted for at the close of the project period, leaving 10,416 cartons (20,832 doses) to be included in analyses. These 10,416 cartons were distributed over 9,170 distinct encounters that are included in the description of THN recipient characteristics.

### Distribution per capita

**Fig 1:**
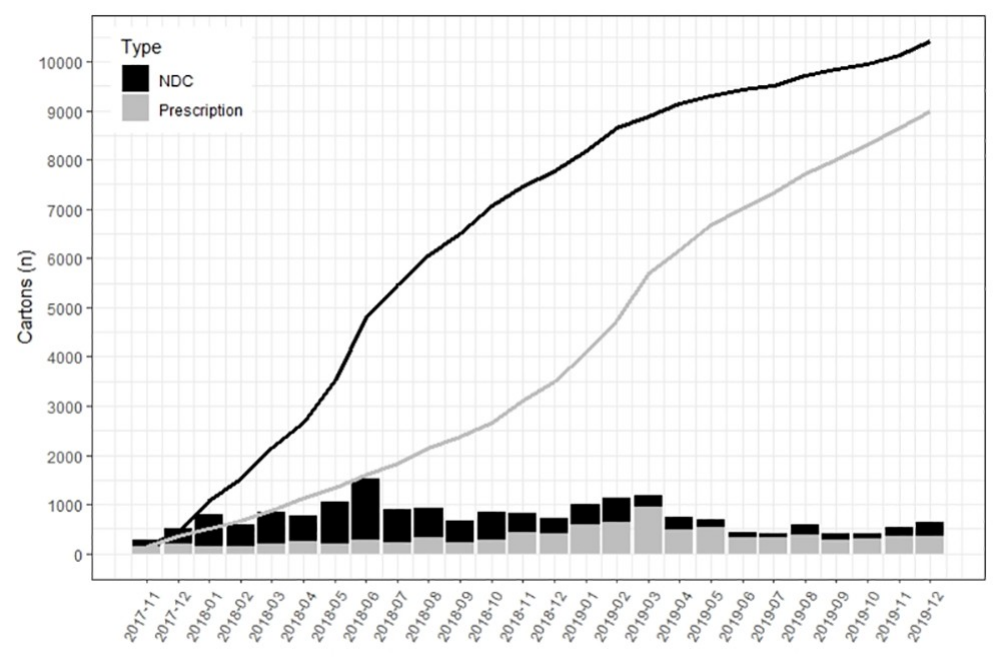
Cumulative and per month take-home naloxone distribution and prescriptions filled. Bars show individual months and the lines are cumulative (cumulative N = 10,416 cartons meeting eligibility criteria; N = 9,158 provider prescriptions for nasal naloxone spray filled within Hamilton County). NDC = Naloxone Distribution Collaborative.

The total distribution of naloxone was 10,416/816,684 county residents, equating to 1,275 cartons/100,000. The average annual rate of distribution was 588/100,000. Fig 1 shows program distribution over time as well as concurrent data for naloxone prescriptions filled. The median number of cartons distributed per month was 349.0 (IQR 185.8–560.8) with the least number of cartons distributed in July 2019 (n = 80) and the most cartons distributed June 2018 (1,266).

### Characteristics of distributions sites and THN recipients

The majority of THN cartons were distributed by syringe exchange service sites and correctional facilities. Table 1 describes all locations in which naloxone was distributed. Table 2 characterizes the individuals known to have received THN. Based on reported characteristics of those that received naloxone, many were at risk for opioid overdose, as evidenced by prior overdose (OD) (23.9% recipients), use of injection drugs (38.7% recipients), prior OUD treatment (32.9% recipients), and wanting the naloxone in case of overdose (26.7% recipients).

**Table 1:** Naloxone carton distribution by venue category^1^.

	n	(%)
**Syringe Services Program**	3,041	(29.2)
**Correctional Facilities**	2,235	(21.5)
**Healthcare Partners**		
*Emergency Departments*	571	(5.5)
*OUD Treatment Facilities*	996	(9.6)
*Pharmacy*	196	(1.9)
*Federally Qualified Health Center*	17	(0.2)
*Urgent Care*	12	(0.1)
*Emergency Medical Services*	11	(0.1)
**Community Outreach**		
*Community Events*	1,949	(18.7)
*Public Health*	298	(2.9)
*Quick Response Teams*^2^	21	(0.2)
*Law Enforcement*	13	(0.1)
**Community Service Organizations**		
*Social Services*	721	(6.9)
*Non-profit*	319	(3.1)
*Faith Based*	14	(0.1)

^1^Distributions of take-home naloxone to individual recipients by site/event type (N = 10,416 THN cartons)

^2^Quick response teams consist of specially trained law enforcement and EMS personnel who respond to overdose calls and link people to treatment as well as provide harm reduction education

**Table 2:** Characteristics of individuals receiving take home naloxone.

	Total n = 9,170	Syringe Services n = 2,979	Correctional Facilities n = 1,982	Healthcare Partners^1^ n = 1,199	Community Outreach n = 2,107	Community Services n = 903
**DEMOGRAPHICS**						
**Age**^2^ (range)	37 (29–48)	36 (30–43)	36 (28–48)	37 (29–48)	38 (28–53)	42 (29–54)
**Gender**^2^ –n (%)						
Male	3,499 (38.2)	1,535 (51.5)	592 (29.9)	395 (32.9)	710 (33.7)	267 (29.6)
Female	4,762 (51.9)	1,294 (43.4)	1,132 (57.1)	548 (45.7)	1,188 (56.4)	600 (66.4)
**Race**^2^ –n (%)						
White	6,436 (70.2)	2,702 (90.7)	993 (50.1)	696 (58.0)	1,458 (69.2)	587 (65.0)
Black	1,396 (15.2)	68 (2.3)	546 (27.5)	213 (17.8)	321 (15.2)	248 (27.5)
Multiracial/Other	1256 (13.7)	190 (6.3)	429 (21.7)	281 (23.5)	296 (14.0)	60 (6.6)
**Ethnicity**^2^ –n (%)						
Hispanic/Latino	218 (2.4)	34 (1.1)	33 (1.7)	26 (2.2)	96 (4.6)	29 (3.2)
**Incarcerated past 30 days**^2^ –n (%)						
Yes	273 (3.0)	125 (4.2)	131 (6.6)	8 (0.7)	7 (0.3)	2 (0.2)
No	2,032 (22.2)	572 (19.2)	298 (15.0)	233 (19.4)	764 (36.3)	165 (18.3)
**OPIOID USE AND OVERDOSE**						
**Prior opioid overdose**^2^ –n (%)						
Yes	2,192 (23.9)	1,587 (53.3)	252 (12.7)	192 (16.0)	106 (5.0)	55 (6.1
No	5,356 (58.4)	1,344 (45.1)	884 (44.6)	721 (60.1)	1,596 (75.7)	811 (89.8
**Multiple Prior Overdoses**^2^ –n (%)^3^						
Yes	1,551 (16.9)	1,163 (39.0)	158 (8.0)	123 (10.3)	71 (3.4)	36 (4.0)
No	580 (6.3)	396 (13.3)	79 (4.0)	62 (5.2)	27 (1.3)	16 (1.8)
**Administered Naloxone**^2^ –n (%)						
Yes	3,105 (33.9)	2,130 (71.5)	372 (18.8)	194 (16.2)	302 (14.3)	107 (11.8)
No	4,467 (48.7)	798 (26.8)	793 (40.0)	713 (59.5)	1,403 (66.6)	760 (84.2)
**Injection Drug Use**^2^ –n (%)						
Yes	3,552 (38.7)	2,663 (89.4)	339 (17.1)	317 (26.4)	162 (7.7)	71 (7.9)
No	3,803 (41.5)	253 (8.5)	785 (39.6)	578 (48.2)	1,431 (67.9)	756 (83.7)
**Past 30 days**^2^ –n (%)						
Yes	2,812 (30.7)	2,508 (84.2)	100 (5.0)	118 (9.8)	77 (3.7)	9 (1.0)
No	598 (6.5)	83 (2.8)	208 (10.5)	176 (14.7)	72 (3.4)	59 (6.5)
**Prior Formal OUD Treatment**^2^ –n (%)						
Yes	3,017 (32.9)	1,963 (65.9)	364 (18.4)	386 (32.2)	201 (9.5)	103 (11.4)
No	4,221 (46.0)	928 (31.2)	732 (36.9)	493 (41.1)	1,348 (64.0)	720 (79.7)
**Would fill prescription**^2^^,^^4^ –n (%)						
Yes	1,865 (20.3)	753 (25.3)	305 (15.4)	146 (12.2)	527 (25.0)	134 (14.8)
No	1,226 (13.4)	510 (17.1)	253 (12.8)	99 (8.3)	260 (12.3)	104 (11.5)
**Reason for Approach**^2^ –n (%)						
Self- request	3,850 (42.0)	597 (20.0)	1,425 (71.9)	642 (53.5)	1,117 (53.0)	69 (7.6)
Staff initiated	4,554 (49.7)	2,164 (72.6)	369 (18.6)	356 (29.7)	902 (42.8)	763 (84.5)
**Reasons wanting naloxone**^2^^,^^5^ –n (%)						
If I overdose	2,445 (26.7)	1,875 (62.9)	145 (7.3)	297 (24.8)	99 (4.7)	29 (3.2)
If friend/family overdoses	2,739 (29.9)	1,655 (55.6)	417 (21.0)	209 (17.4)	337 (16.0)	121 (13.4)
If I witness an overdose	4,857 (53.0)	1,610 (54.0)	703 (35.5)	406 (33.9)	1,532 (72.7)	606 (67.1)
To have on hand	1,413 (15.4)	232 (7.8)	208 (10.5)	233 (19.4)	451 (21.4)	289 (32.0)

All variables are reported as number and proportion except for age which is reported as median and interquartile range. Since efforts to confirm identity were not allowed to interfere with distribution, data is reported at the encounter level; unique individuals could have had more than one encounter. Some individuals received more than one carton. Whether cartons were used and whether used by the individual who received them is unknowable; secondary distribution through social networks was anecdotally common

^1^Healthcare partners does not include the 571 distribution encounters (573 cartons) from the ED which only had demographic data available: age = 34.0 (29.0–42.0); gender = 384 (67.3) Male, 187 (32.7) Female; race = 500 (87.6) White, 61 (10.7) Black, 3 (0.5) Hispanic/Latino, 1 (0.2) Multiracial, 5 (0.9) Other, 1 (0.2).

^2^Data missing as follows: age (n = 726); gender (n = 909); race/ethnicity (n = 1038); incarnated (n = 6,865); prior overdose (n = 1,622); multiple prior overdose (n = 7,039); administered naloxone (n = 1,598); injection drug use (n = 1,815); past 30 days injection drug use (n = 5,760); prior OUD treatment (n = 1,932); would fill a naloxone prescription (n = 5,600); reason for approach/encounter initiation (n = 766); reasons wanting naloxone (n = 750)

^3^This question was only asked of those who answered yes to a prior opioid overdose

^4^Theoretically would be willing to fill a prescription for naloxone (i.e. naloxone was provided at encounter and a prescription was not offered). Question added 01/08/2018, N = 8,691encounters thereafter used in denominator calculation.

^5^More than one reason could be selected (i.e. not mutually exclusive)

### Opioid-related overdose deaths

**Fig 2:**
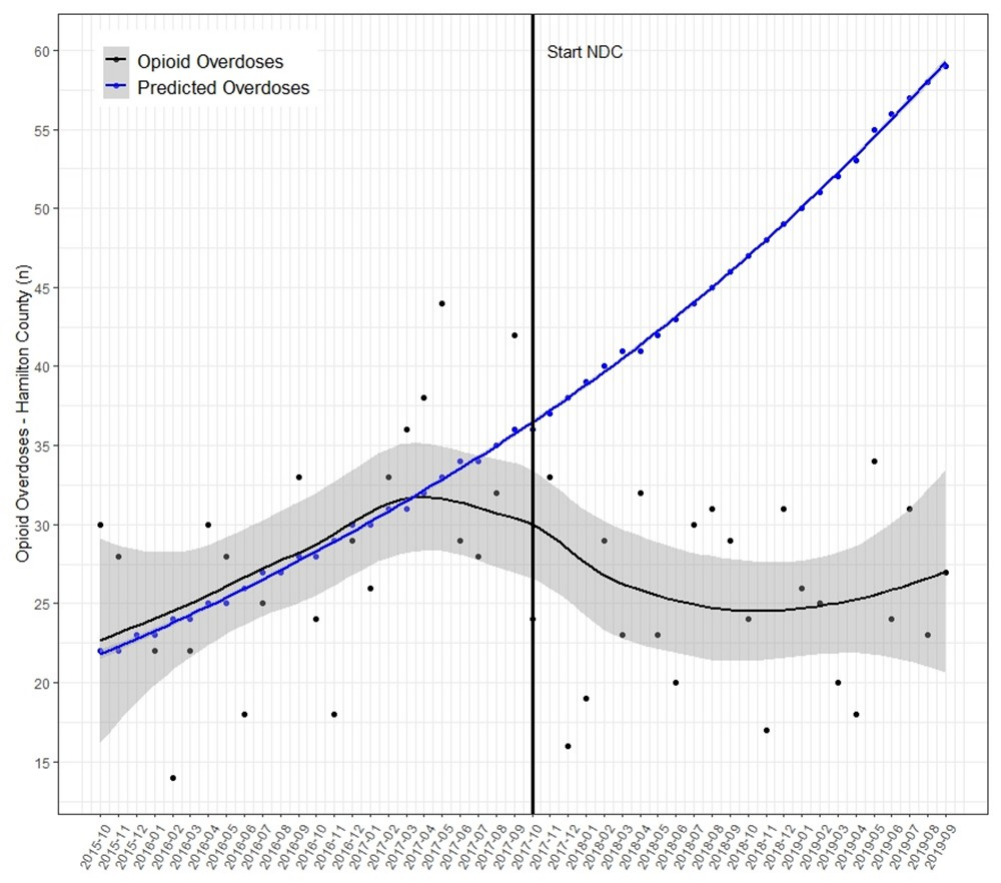
Opioid overdose deaths. Each dot is the number of overall opioid-overdose deaths of county residents that month, the black line is the LOESS smoothed curve (shaded area is 95% CI), the blue dots are the estimated value for each month, and the blue line is the negative binomial regression trendline.

The absolute number of opioid overdose deaths decreased from 679 to 609 when comparing the two year periods before and after the start of the THN program, equating to a 10.3% (95% CI 8.1–12.9) absolute decrease. Median monthly overdose mortality in the two years before (28 persons, 95% CI 25.0–31.0) and during (24.5, 95% CI 20.0–26.0) the program did not differ significantly. Fig 2 depicts the trend in OOD rate for Hamilton County. Given the trend during the two years prior to the start of the naloxone distribution efforts associated with this project, the total number of predicted deaths for Hamilton County was 1806, whereas the actual observed deaths was only 1288.

Table 3 details the changes in OOD for other counties in the state during the same time periods. Counties included are those immediately surrounding the county of interest, as well as other large urban counties. This demonstrates that there was a decrease in OOD throughout the state, with Franklin County as an outlier experiencing increased OOD.

**Table 3:** Change in total number of persons who died due to an opioid overdose.

	Oct 2015-Sept 2017	Oct 2017-Sept 2019	Difference	% Change (95% CI)
Ohio—ALL counties	7312	6299	-1013	-13.9 (-13.1 to -14.7)
Ohio, excluding Hamilton County	6633	5690	-943	-14.2 (-13.3 to -15.1)
Hamilton County	679	609	-70	-10.3 (-8.2 to -12.9)
**Surrounding Counties**				
Butler	420	316	-104	-24.8 (-20.8 to -29.2)
Clermont	173	145	-28	-16.2 (-11.2 to -22.7)
Warren	123	77	-46	-37.4 (-29.0 to -46.6)
**Large Urban Counties**				
Cuyahoga	956	766	-190	-19.9 (-17.4 to -22.6)
Franklin	606	862	256	42.2 (38.3 to 46.3)

## Discussion

This evaluation characterizes a regional take-home naloxone distribution effort that was notable for its scope and prioritization of generalized and rapid distribution over selected population targeting and rigorous documentation. The annual per capita naloxone distribution of 588/100,000 surpasses our calculations of previously reported programs during similar time periods, nearly four times the reports from Norway of 152.4/100,000 [25, 26], and slightly outpacing programs in Canada at 495/100,000 [27], and New England at 441/100,000 in Massachusetts and 548/100,000 in Rhode Island [28]. Of these other distribution programs, only the one in Canada had a general distribution effort similar to ours, while the other programs focused on targeted populations for distribution. This high rate of distribution demonstrates that, given adequate resources, population demand, and community-wide collaboration, it is possible to markedly increase the presence of THN within a local community. Moreover, our primary analysis is likely an underestimate of the achieved distribution, since it only included those cartons known to have been received by a person and not the additional cartons provided to organizations who did not provide complete data on the final disposition of the supply. It also does not account for the use of naloxone provided through this project to first responders who may not have otherwise had the means to supply this life saving medication for use in the field. Finally, we used cartons rather than doses as our unit of analysis. Each carton contained two doses, which may have been divided between people or used at different time points.

A key question for any intervention is which populations to select, with the intuitive priority being those most in need. However, THN is complicated by the fact that the victim cannot self-administer the medication, so the population in need is defined by likelihood of encountering a person who has overdosed rather than one’s own risk of overdose. This includes those known socially to a person at-risk. However, it also includes the much larger population of potential lay person responders who may coincidentally happen upon an overdose victim. Arguments in favor of a more selected distribution approach include: i) American Society of Addiction Medicine prioritization of those who are at highest risk for opioid overdose and people who are close to them [29], ii) the preponderance of OODs that happen in a private setting [25, 30], iii) modeling indicating that distribution at sites serving high-risk individuals has the largest impact on mortality [12], and iv) likelihood of increased THN cost-effectiveness when targeting high-risk populations [31, 32]. Nonetheless, a focus on the potential of general bystander response drove the more indiscriminate and generalized approach of this project. The health department did attempt to emphasize at-risk and disadvantaged populations where possible, evidenced by the higher distribution numbers seen at sites such as the syringe service programs and correctional facilities.

Program success was predicated on strong innate demand from at least some portions of the community. Overall, there were 97 sites spanning multiple sectors that received naloxone for distribution and/or to have on hand for bystander administration. At the close of the program, the health department was still receiving new requests for naloxone, and there were a variety of anecdotal reports all consistent with significant demand: i) clients seeking naloxone on more than one occasion, ii) secondary distribution, whereby the client served would report giving part of their supply to others in their community, and iii) emergency physician reports of more patients receiving bystander naloxone prior to EMS arrival. It is possible that community acceptance of naloxone in this experience was so high due to the availability of pre-packaged nasal naloxone spray, which has been found to have improved usability and quicker time to administration when compared to injectable or atomized naloxone [33, 34]. General acceptance of naloxone is increasing, as shown by willingness of lay people to respond to an overdose and administer naloxone after being alerted by a smartphone application [35].

There were certain trends noted among demographics of those who accepted naloxone through this program. A majority of people were in their 4^th^ decade of life. This aligns with the age groups who experiences the highest rate of opioid overdose deaths [36]. A majority of recipients were white, with a significant predominance at the syringe service sites. There is a known racial disparity in those that access syringe service sites, with non-whites not as likely to utilize these services regardless of distance to sites [37, 38]. These findings reinforce prior work and highlight the need to improve outreach to non-white communities to ensure they are aware of and feel safe accessing these services. Of note, women were more likely to accept naloxone at community events. There is evidence demonstrating that older white men are more likely to receive aid when suffering from a medical emergency, but no reports on characteristics regarding the bystanders that are rendering the aid [39, 40]. Determining who renders aid and whether they may be a disparity based on sex, race or other characteristics deserves further exploration.

Not surprisingly, those who received naloxone from syringe services sites were more likely to have experienced an overdose, use intravenous drugs or have administered naloxone in the past compared to those who received naloxone at other sites. It is encouraging to see that such a large number of people with no prior naloxone experience were accepting of the medication at other sites. Of note, a vast majority of those that accepted naloxone at community events reported wanting it in case they witnessed an overdose, indicating that it was more likely to be used on someone other than themselves or a family member.

Despite health systems helping to fund the program, there was low distribution numbers from the health care sector compared to within the community. Operational barriers to participation in naloxone distribution initiatives has been previously documented [27, 41]. and provider perceptions and attitudes are a barrier for naloxone specifically [42–45]. Over the project period, there was an increase in the number of naloxone prescriptions filled at local pharmacies, suggesting increased prescribing, but it is unclear from what source these prescriptions originated. Of note, the degree to which prescriptions can functionally substitute for direct distribution is unknown. The summary of existing literature by Irvine *et al*. found that increasing prescriptions for naloxone had a much smaller impact on probability that it was available for use at a witnessed OOD than distribution from a pharmacy or through the community [19]. We did find that of those directly receiving naloxone who responded to the survey question, over half said they would theoretically be willing to fill a prescription. Further work should clarify the relative importance of healthcare sector participation, direct distribution versus prescription, stationary naloxone placement akin to automatic external defibrillators [46], and for distribution, the role of direct versus mail delivery [47].

While simply accomplishing distribution at this scale is achievement of interest, we do not know the true impact of this program or the degree to which available resources could have been used more appropriately in other ways. The observed rate of OOD did not differ substantially before versus during the naloxone distribution period. This is consistent with prior findings by Zang *et al*. demonstrating no association between the rate of naloxone distribution and rate of OOD [28], and modeling by Linas *et al*. suggest naloxone distribution alone is unlikely to significantly decrease OOD without concurrent engagement in evidence-based OUD treatment that includes medication [48]. A program in Canada has targeted distribution to sites where staff may have the need to respond to an opioid overdose, such as shelters [49]. The experience there has been that hundreds of naloxone doses are utilized each year and a majority of staff report feeling comfortable with administration of the medication. A limitation of this study is that we have no other data to determine whether THN distributed by the program evaluated in this study was used, who used it, whom it was used on or whether the use was beneficial. Prior studies have found that 10%-27% of distributed naloxone is used for reversal of opioid overdose, which is likely undercounted due to rates of lost to follow-up [25, 27, 41]. How usage frequency varies depending on approach to distribution is unknownTo date, there is little to no data to empirically guide distribution programs in balancing the tradeoffs between appropriateness of distribution and rate of distribution.

Although there was a decrease in number of opioid deaths after the naloxone distribution program was implemented, this was not noted to be statistically significant. In addition, this decrease was seen in the surrounding counties as well, where there was no known significant quantities of naloxone being distributed. The decrease in overdose deaths is attributed in part to the presence of carfentanil in the drug supply in 2016–2017 [50].

This retrospective study design, with neither rigorous prospective data collection nor group comparison, would not support causal inference even if we had observed a larger change in OOD. Moreover, there are a host of unmeasured factors plausibly contributory to changes in either naloxone use or OOD mortality. Most important among these is the rate of non-fatal opioid-related overdose, as the proportion of overdoses resulting in death is a more appropriate measure of naloxone impact than is the absolute frequency of OOD over time. Unfortunately, there is no readily available and reliable measure of non-fatal overdoses. Emergency medical services (EMS) may not be called, especially if bystander naloxone is administered, and EMS may not transport victims of non-fatal OOD to the emergency department (ED), often because of patient refusal.

## Conclusions

Massive and rapid naloxone distribution to lay bystanders is feasible. Even large-scale take-home naloxone distribution may not substantially reduce opioid overdose mortality rates.

## Supporting information

S1 Appendix These forms were used to collect information from those receiving naloxone kits. (DOCX)
