# Supplementary material for: Evaluation of a large-scale health department naloxone distribution program: Per capita naloxone distribution and overdose morality
Source: PLoS One. 2023 Aug 11;18(8):e0289959. doi: 10.1371/journal.pone.0289959 (PMC10420337; doi:10.1371/journal.pone.0289959)
Supplement: S1 Appendix — (DOCX) [file pone.0289959.s001.docx]

**Appendix A:** Narcan Distribution Data Collection Form

| **Last Name*: _____________________________** | | **First Name*: ___________________________** | |
| --- | --- | --- | --- |
| **Date of Birth** (mm/dd/yyyy)**:** __ __ / __ __ / __ __ __ __  OR  **Age** (years)**:** ______   ☐ Not available | | | |
| **Do you consider yourself to be male or female?** ☐ Male   ☐ Female   ☐ Not available | | | |
| **What race(s) and ethnicity do you consider yourself (check all that apply):** | | | |
| ☐ White   ☐ Black/African American   ☐ Hispanic/Latino   ☐ Asian   ☐ Other: _____________   ☐ Not available | | | |
| **Street Address*: ___________________________________________________________________** | | | |
| ☐ Typical address not applicable (for example, homeless) | | | |
| ***If typical address not applicable, please include information available, such as intersection, neighborhood, etc. (for example, the person sleeps downtown near corner of 5^th^ and Vine)*** | | | |
| **City*: ____________________** | **State*: ___________________** | | **Zip*: __ __ __ __ __** |

| **Intended use for Narcan (check all that apply):** | | | ☐ If I overdose | ☐ If friend/family member overdoses |
| --- | --- | --- | --- | --- |
|  |  |  | ☐ If I see someone overdose | ☐ For location to have on hand |
| **Yes** | **No** | **Have you ever:** | | |
| ☐ | ☐ | Administered (used) Narcan or Naloxone on someone overdosing on heroin or other opioid? | | |
| ☐ | ☐ | Overdosed yourself on heroin or other opioid? | | |
| ☐ | ☐ | ***If yes,*** *have you overdosed on heroin or other opioid more than once?* | | |
| ☐ | ☐ | Used IV drugs? | | |
| ☐ | ☐ | ***If yes,*** *have you used IV drugs in the past 30 days?* | | |
| ☐ | ☐ | Been in a formal treatment program (other than AA, NA, or other peer support groups)? | | |
| ☐ | ☐ | Have you been released from a correctional facility in the past 30 days? | | |
| ☐ | ☐ | If you were given a prescription for Narcan (instead of receiving today), would you fill prescription at pharmacy? | | |

****Portion below to be filled out by instructor/distributor ****

| **Narcan given to individual?*** ☒ Yes   ☐ No | **Trained on Narcan Administration?*** ☒ Yes   ☐ No |
| --- | --- |
| ***If Narcan given:* Number of Narcan cartons given* __ __** | |
| **Date given*** (mm/dd/yyyy)**:** __ __ / __ __ / __ __ __ __ | |
| **Lot # * __________________** | **Expiration Date*** (mm/dd/yyyy)**:** __ __ / __ __ / __ __ __ __ |
| **Interaction type:** ☐ Staff initiated Narcan offer   ☐ Individual asked for Narcan | |
